# Natural Radioactivity in Soil and Water from Likuyu Village in the Neighborhood of Mkuju Uranium Deposit

**DOI:** 10.1155/2013/501856

**Published:** 2013-05-28

**Authors:** Najat K. Mohammed, Mohamed S. Mazunga

**Affiliations:** Department of Physics, University of Dar es Salaam, P.O. Box 35063, Dar es Salaam, Tanzania

## Abstract

The discovery of high concentration uranium deposit at Mkuju, southern part of Tanzania, has brought concern about the levels of natural radioactivity at villages in the neighborhood of the deposit. This study determined the radioactivity levels of 30 soil samples and 20 water samples from Likuyu village which is 54 km east of the uranium deposit. The concentrations of the natural radionuclides ^238^U, ^232^Th, and ^40^K were determined using low level gamma spectrometry of the Tanzania Atomic Energy Commission (TAEC) Laboratory in Arusha. The average radioactivity concentrations obtained in soil samples for ^238^U (51.7 Bq/kg), ^232^Th (36.4 Bq/kg), and ^40^K (564.3 Bq/kg) were higher than the worldwide average concentrations value of these radionuclides reported by UNSCEAR, 2000. The average activity concentration value of ^238^U (2.35 Bq/L) and ^232^Th (1.85 Bq/L) in water samples was similar and comparable to their mean concentrations in the control sample collected from Nduluma River in Arusha.

## 1. Introduction

The radioactivity level from the natural radionuclides is termed as background radiation which will depend on the amount of the radioactive materials in the environment. The background radiation can be high if the environment is polluted either from man-made or natural activities. It can also be high in regions with deposit of mineral resources such as uranium ores and phosphate [1]. Materials from the deposit may be brought to the surface soil through processes such as weathering of rocks and soil formation. They can also leach into the groundwater system, contaminate it, and lead to pollution far away from the source. 

In Tanzania, high concentrations of uranium deposits of up to 464 ppm have recently been reported at Mkuju in Namtumbo area at Ruvuma region. This has brought concern about the level of natural radioactivity in the soil and drinking water at villages in the neighborhood of the deposit. This is because there are reports in the literature which indicate high radioactivity levels in regions near uranium deposits [2, 3]. Rabesiranana et al. (2008) in Madagascar and Sartandel et al. (2009) in India reported enhanced level of ^238^U and ^232^Th in the soil around uranium deposits as compared to the world average [2, 4]. Enkhbat et al. (2008) reported high activity concentration of ^226^Ra, ^232^Th, and ^40^K in soil around Gurvanbulag uranium deposit in the eastern part of Mongolia [3].

The present study has determined concentration levels of the ^238^U decay series and the ^232^Th decay series as well as ^40^K in soil and water from Likuyu village which is 54 km east of the Mkuju uranium deposit in Tanzania. The paper will use ^238^U and ^232^Th to indicate the concentrations of these decay series. The data will offer useful and necessary information in the monitoring of environmental contamination which will provide appropriate and better protection guidelines to the public. 

## 2. Material and Methods

### 2.1. Study Area

The Likuyu-Seka-Maganga village is at Namtumbo district in Ruvuma region southern Tanzania. The village is situated at longitude 36°16′ east and latitude 10°19′ south of the Equator. It is near to the Undendeule Forest Reserve (UFR) at the south and the Selous Game Reserve (SGR) at the north. The Likuyu village is situated at about 54 km east of the Mkuju uranium deposit. The only accessible road to the Mkuju uranium deposit is through Likuyu village.

The ambient temperature at the Likuyu village varies from minimum of 18.8°C to a maximum of 28.2°C. The wind blows from the Mkuju uranium deposit through the Likuyu village in the month of December to February with speed of up to 11.6 m/s. Two rivers, which are Mkuju River and Kilowelo River, flow from the Mkuju uranium deposit to the northern and southern parts of the Likuyu village, respectively. The water from these rivers is domestically used by the people of the Likuyu village. The village has a total population of about 10,000 people settled in eight hamlets, seven of which lie along the road to the Mkuju uranium deposit (Mantra, 2010) [5]. 

### 2.2. Sampling Methodology

The village was divided into three sampling zones: the northern, the central, and the southern zones. Ten samples of soil were collected randomly from each sampling area to make a total of 30 samples of soil. The samples were collected at 0–5 cm depth level as reported elsewhere [6]. The collected samples were then placed in labeled polythene bags and transferred to the TAEC laboratory for preparation and analysis.

Twenty water samples were collected from the two rivers passing through the village. The samples were filled in a one liter acid precleaned polyethylene container to avoid wall absorption (IAEA, 1989) and were wrapped using insulation tape to avoid spill during transport of the samples from sampling point to the TAEC laboratory for preparation and analysis [7]. 

### 2.3. Sample Preparation

In the laboratory, the soil samples were sieved by a 2 mm mesh to remove larger objects and then ground using mortar and pestle to fine powder in order to have the same matrix as the reference sample. After that, the samples were dried in an oven at a temperature around 45–50°C for several hours until constant weights were attained and then placed in desissators to avoid moisture absorption as described elsewhere [8]. Samples of about 500 g each were packed in Marinelli beaker of about 500 cm^3^ volume and sealed using silicon and plastic tapes for air tight. The samples were left for a minimum of 28 days for radioactive secular equilibrium between ^222^Rn-radon gas and its decay products (^214^Pb, ^214^Bi, and ^226^Ra), from the ^238^U decay series to be acquired.

The activity of ^238^U was determined using the gamma-lines of ^226^Ra (186 keV), ^214^Pb (295 keV and 351 keV), ^214^Bi (1238 keV and 1378 keV), ^228^Ac (338 keV, 911 keV, 965 keV, 969 keV), and ^208^Tl (860 keV). The gamma-lines of 609.3 keV, 1120 keV (from ^238^U series), 583.2 keV, 727.3 keV, and 795 keV (^232^Th series) were omitted from the analyses as sensitivity analysis has shown elsewhere that they are affected by coincidence summing [9]. The ^40^K was measured from its gamma line energy of 1460.8 keV.

### 2.4. Gamma Spectrometry

A lead-shielded coaxial high-purity germanium detector (HPGe) of relative efficiency 51% and resolution of 2.1 keV for 1332 keV ^60^Co gamma-ray source was used for low level counting of the samples. The detector chamber was shielded with three layers of copper, cadmium, and lead of 30 mm, 3 mm, and 100 mm thick, respectively. The *γ*-ray energy calibration was performed daily using standard radiation sources of ^133^Ba, ^109^Cd, ^54^Mn, ^57^Co, ^60^Co ^65^Zn, ^22^Na, and ^137^Cs. Efficiency calibration was determined using In Situ Object Counting-system (ISOCS) of Genie 2000 software. 

The activity concentration (in Bq kg^−1^), *A*_*Ei*_ of a radionuclide *i* and for a peak of energy *E*, in a samples was calculated using the following formula [10]:

(1) $$\begin{array}{c} A_{Ei}=\frac{N_{Ei}}{\gamma_{d}\times \varepsilon_{E}\times m_{s}\times T}, \end{array}$$

where *N*_*Ei*_ is the net peak area at energy *E*, *ε*_*E*_ is the detection efficiency at the energy *E*, *T* is the counting live time in seconds, *γ*_*d*_ is the gamma ray yield per disintegration of the specific radionuclide for a transition at energy *E*, and *m*_*s*_ is the mass of the dry weight in kg of the measured sample.

The IAEA soil 375 was used as a standard reference material to evaluate the precision and accuracy of the gamma results. The standard reference material was counted at the same time as used for counting the samples (8 hours), and then its activity at various energies was calculated and compared with the certified value after correction for the decay by considering the date (December 31st, 1991) provided in the data sheet. As shown in Table 1, the experimental concentration values agreed well with the recommended values within 10% accuracy.

## 3. Results and Discussion

### 3.1. Soil Samples

The average natural radionuclide activity concentrations in the soil samples collected from the three zones at the Likuyu village are reported in Table 2. Specific activities concentrations of ^238^U, ^232^Th, and ^40^K are reported in Bq kg^−1^ dry weight. 

The lowest mean values of all three radionuclides were found in samples from the central part of the village. The northern zone had the highest concentrations of ^238^U and ^40^K, which were each 1.2 times higher than their values determined in samples from the central zone. Samples from the southern zone had the highest mean value of ^232^Th. This value was 1.3 times higher than the mean value obtained in samples collected from the central zone. Rivers Mkuju and Kilowero flow from the Mkuju uranium deposit to the northern and southern zones, respectively. Higher values of radionuclides in samples from these zones compared to the values determined in samples from central zone might indicate the rivers transportation of radionuclides from Mkuju uranium deposit to the village.

The mean activity concentrations of natural radioactivity found in the soil in the present study were compared with the range and average of natural radioactivity concentration levels in soils of eight countries reported by UNSCEAR (2000) (Table 3) [11]. The mean concentration of ^238^U found in this study was found to be higher than its mean concentrations in soil of 75% of the countries reported by UNSCEAR (2000). The value is also higher than the world average value; however, it lies within the ranges of ^238^U revealed in soils of all countries in the UNSCEAR report (UNSCEAR, 2000). The mean concentration of ^232^Th obtained in the study is comparable to the world average value but lower than the mean concentrations in 63% of the countries reported by UNSCEAR (2000) [11]. 

### 3.2. Water Samples

The average natural radionuclide activity concentrations in the water samples collected from the two rivers in Likuyu village are reported in Table 4. Specific activities concentrations of ^238^U, ^232^Th, and ^40^K are reported in Bq L^−1^. 

As Table 4 shows, samples from W1 and W2 had similar concentration of each radionuclide ^238^U, ^232^Th, and ^40^K. The concentration of ^238^U and ^232^Th was similar to the concentration in the control sample collected from Nduluma River in Arusha, whilst the mean activity level of ^40^K was 5 times higher than that of the control sample.

**Figure 1:**
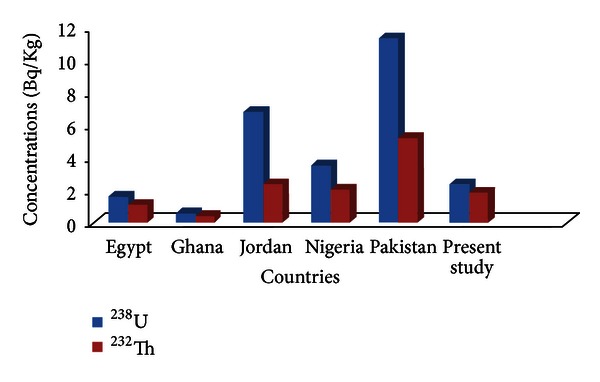
The average radioactivity concentrations values of water samples obtained in the present study compared with studies conducted elsewhere in the world.

Figure 1 compares the mean concentration of radionuclides from the two rivers in this study with those obtained in rivers of 5 countries reported in the literature.

The mean concentration of ^238^U from the two rivers in Likuyu was similar and approximately 4 times higher than the activity value reported by Faanu et al. (2011) in a river at Tarkwa Gold mine in Ghana [12]. This value was only 1.5 times higher than the value reported by El Arabi et al. (2006) in natural water resources from Elba protective area in Egypt [13]. 

The mean value reported in this study was also 3 times lower than the value reported in water samples collected from a river in Jordan. The mean concentration level of ^232^Th found in this study was lower than the mean activity reported in Pakistan, Jordan, and Nigeria, but slightly higher than the value reported in Ghana and Egypt. 

## 4. Conclusion

This study determined the activity concentrations of natural occurring radionuclides in soil and water samples collected at Likuyu village which is 54 Km east of the Mkuju uranium deposit in southern Tanzania. The measurements were carried out using low level gamma ray spectrometry technique at the TAEC laboratory in Arusha.

The mean concentration of ^238^U (51.7 Bq/Kg) soil analyzed in this study was found to be higher than its mean concentrations in soil of 75% of the countries reported by UNSCEAR (2000). The value is also higher than the world average value; however, it lies within the ranges of ^238^U shown in soils of all countries in the UNSCEAR report (UNSCEAR, 2000). The mean concentration of ^232^Th obtained in the study is comparable to the world average value but lower than the mean concentrations in 63% of the countries reported by UNSCEAR (2000). In this study, the highest mean values of all three radionuclides were found in samples from northern and southern parts of the village, which might be influenced by the flow of rivers Mkuju and Kilowero from the Mkuju uranium deposit to the village. 

The results of radioactivity concentrations in the water sample obtained in this study were comparable with the concentration values of natural radioactivity reported in the literature. Moreover, their concentrations were similar to those obtained in the control sample collected from Nduluma River in Arusha. It is recommended to consider seasonal variations in determining radioactivity at Likuyu village. This is because during rainy season rivers might have elevated concentration of radionuclides due to the soil erosion at Mkuju Uranium Deposit. 

## Figures and Tables

**Table 1:** The Standard reference values and experimental activity values of the IAEA reference soil 375 (Bq/kg ± SD).

Radionuclides	Energy (keV)	Experimental activity concentration	Certified reference values
^ 226^Ra	186.5 keV	20.54 ± 0.9	20.0 ± 0.9
^ 214^Pb	295.2	19.46 ± 0.8	19.96 ± 0.8
	351.92	20.61 ± 1.1	19.96 ± 0.5
^ 214^Bi	1764	20.94 ± 0.8	19.96 ± 0.4
	2477.7	22.46 ± 1.2	19.96 ± 0.9
^ 40^K	1460	499.93 ± 2.1	423.40 ± 0.2
^ 228^Ac	338.5	21.58 ± 1.5	20.46 ± 0.3
^ 208^TI	860.4	22.20 ± 1.1	20.46 ± 0.3
	2614.5	22.44 ± 1.7	20.46 ± 0.6
^ 228^Ac	910.7	22.29 ± 0.7	20.46 ± 0.5
	968.5	23.69 ± 0.9	20.46 ± 0.5

**Table 2:** The average activity concentration of radionuclides in the collected soil samples (Bq/kg ± SEM).

Soil samples (*N* = 10)	Activity concentration		
	^ 238^U	^ 232^Th	^ 40^K
Northern zone	57 ± 5	36 ± 3	616 ± 113
Southern zone	52 ± 4	41 ± 3	558 ± 10
Central zone	46 ± 3	32 ± 3	519 ± 7

Average	52 ± 4	36 ± 3	564 ± 10

**Table 3:** Comparison of natural radioactivity levels in the soil of Likuyu village with those in other countries as given in UNSCEAR (2000) [11].

Countries	Activity concentration (Bq/kg)		
	^ 238^U	^ 232^Th	^ 40^K
Malaysia	66 (49–86)	82 (63–110)	310 (170–430)
China	32 (2–440)	41 (1–360)	440 (38–760)
Egypt	37 (6–120)	18 (2–96)	320 (29–650)
Algeria	30 (2–110)	25 (2–140)	370 (66–1150)
India	29 (7–81)	64 (14–160)	400 (38–760)
Portugal	49 (26–82)	51 (22–100)	840 (220–1230)
Hong Kong SAR	84 (25–130)	95 (16–200)	530 (80–1100)
United States	35 (4–140)	35 (4–130)	370 (100–700)
World average	30 (16–110)	35 (11–64)	400 (140–850)
Present study	**52**	**36**	**564**

**Table 4:** The average activity concentration of radionuclides in the collected water samples (Bq/L ± SEM).

Water samples	Mean radioactivity concentration		
	^ 238^U	^ 232^Th	^ 40^K
Mkuju River (W1)	2.5 ± 0.4	1.9 ± 0.2	11.0 ± 2.0
Kilowelo River (W2)	2.2 ± 0.3	1.8 ± 0.1	9.2 ± 1.2
Control sample	2.1 ± 0.3	1.7 ± 0.2	2.0 ± 0.8
